# Microplastic contamination in surface water and sediments of Lake Canaçari, Central Amazon, during the dry season

**DOI:** 10.1007/s10661-026-16079-2

**Published:** 2026-10-19

**Authors:** Gabriel dos Anjos Guimarães, Rayane Vasconcelos da Fonseca, Beatriz Rocha de Moraes, Rômulo Augusto Ando, Isreele Jussara de Azevedo Rodrigues, Gustavo Frigi Perotti, Bruno Sampaio Sant’Anna, Gustavo Yomar Hattori

**Affiliations:** 1 Universidade Federal Do Pará, Instituto de Geociências, Avenida Augusto Corrêa S/NGuamá, Belém, Pará 66075-110 Brazil; 2 Universidade Federal Do Amazonas, Instituto de Ciências Exatas E Tecnologia, Rua Nossa Senhora Do Rosário, 3863, Tiradentes, Itacoatiara, Amazonas 69103-128 Brazil; 3 Programa de Pós-Graduação Em Botânica, Instituto Nacional de Pesquisas da Amazônia, Av. André Araújo, 2936, Petrópolis, Manaus, Amazonas 69067-375 Brazil; 4 Departamento de Química Fundamental, Universidade de São Paulo, Instituto de Química, Av. Professor Lineu Prestes, 748–B4T, Butantã, São Paulo, São Paulo 05508000 Brazil

**Keywords:** Amazon, Aquatic pollution, Fishing activity, Human activities, Pigments, Plastic fibers

## Abstract

Contamination by microplastics is an emerging environmental problem that is widely distributed across continental aquatic environments and may cause ecological impacts. This study evaluated the occurrence, concentration, and chemical and morphological characteristics of microplastics in surface waters and sediments from Lake Canaçari, comparing two areas with different land-use patterns within a floodplain system in the Central Amazon. Microplastics were detected in all sampled areas. In surface water, concentrations ranged from 0 (no microplastic particles detected in one sample) to 1,000 microplastics·m⁻^3^ (405 ± 298 microplastics·m⁻^3^), while in sediments, they ranged from 7 to 307 microplastics·kg⁻^1^ (77 ± 104 microplastics·kg⁻^1^). No significant differences in microplastic concentrations were observed between fishing and human-settlement areas. The particles were predominantly blue fibers (< 1,000 μm). Among the spectroscopically analyzed particles, polyethylene was the most frequently identified polymer, while copper phthalocyanine pigments, including Pigment Blue 15 and Pigment Green 7, were also detected by FTIR and/or Raman spectroscopy. Overall, the results suggest that Lake Canaçari may act as a transport pathway, redistribution zone, and retention compartment for microplastics; however, seasonal sampling is needed to confirm how these processes vary throughout the hydrological cycle. These results reinforce the role of Amazonian floodplain systems in the regional dynamics of microplastics and highlight the need for monitoring and management strategies that incorporate seasonal hydrological connectivity at the basin scale.

## Introduction

In recent years, microplastics have emerged as one of the main environmental concerns at a global scale, due to its widespread distribution and persistence in ecosystems (Ibrahim et al., 2025). These contaminants have already been recorded in several environmental compartments, including marine, freshwater, and terrestrial environments, as well as in living organisms. In the Amazon region, recent studies have investigated the presence of microplastics in different environments (Melo et al., 2026), with emphasis on their ingestion by aquatic organisms, highlighting the increasing incorporation of these pollutants into trophic chains.

The Amazon River basin, the largest fluvial system in the world in terms of length, drainage area, and discharge, also stands out for its extraordinary biodiversity (Costa et al., 2021). However, this system has been increasingly pressured by anthropogenic activities and is now considered an important pathway for the transport of plastic waste to the ocean. Model-based estimates suggest that the Amazon River may discharge approximately 38,000–182,000 tons of plastic annually; however, these estimates are subject to considerable uncertainty due to limitations in available data and model assumptions (Lebreton et al., 2017; Giarrizzo et al., 2019). Such plastic inputs may contribute to the formation and dispersion of microplastics in the region (Morais et al., 2024).

Microplastics are defined as solid particles smaller than 5 mm, occurring in the form of fragments, fibers, films, or foams (Lusher et al., 2020). These particles originate from diverse sources, including urban and industrial activities, inadequate waste management, domestic effluents, and the degradation of plastic materials (Feng et al., 2025). Additionally, specific sources such as abandoned or inadvertently discarded fishing nets also contribute significantly to the generation of microplastics, as they undergo fragmentation through physical and chemical processes (Ramos et al., 2025). Owing to their persistence and ability to adsorb contaminants, microplastics pose potential risks to aquatic biota and human health (Uzun et al., 2022).

Despite advances in research, important knowledge gaps still remain regarding the occurrence and dynamics of microplastics in Amazonian freshwater environments, especially in floodplain lake systems. Lake Canaçari, located on the left bank of the Amazon River, is classified as an open and relatively homogeneous floodplain lake, whose hydrological characteristics favor particle accumulation during hydrological cycles. Recent evidence has already demonstrated the presence of microplastics in siluriform fish from this lake (Azevedo et al., 2024), indicating the occurrence of these particles in the system and their potential transfer through the food chain.

Given this context, it is essential to quantify and characterize microplastics in the surface water and sediments of Lake Canaçari. In addition to helping fill knowledge gaps, this investigation is essential for understanding potential ecological impacts and risks to human health, especially considering the dependence of riverside populations on local fishery resources. The present study aimed to quantify and characterize microplastics in the surface water and sediments of Lake Canaçari, in relation to the hydrological dynamics of the region, by comparing levels of microplastics between fishing areas and areas with human settlements. The results are intended to support environmental management and conservation actions for Amazonian ecosystems, contributing to the protection of a region of high socio-environmental importance.

## Materials and methods

### Sample collection and processing

**Fig. 1:**
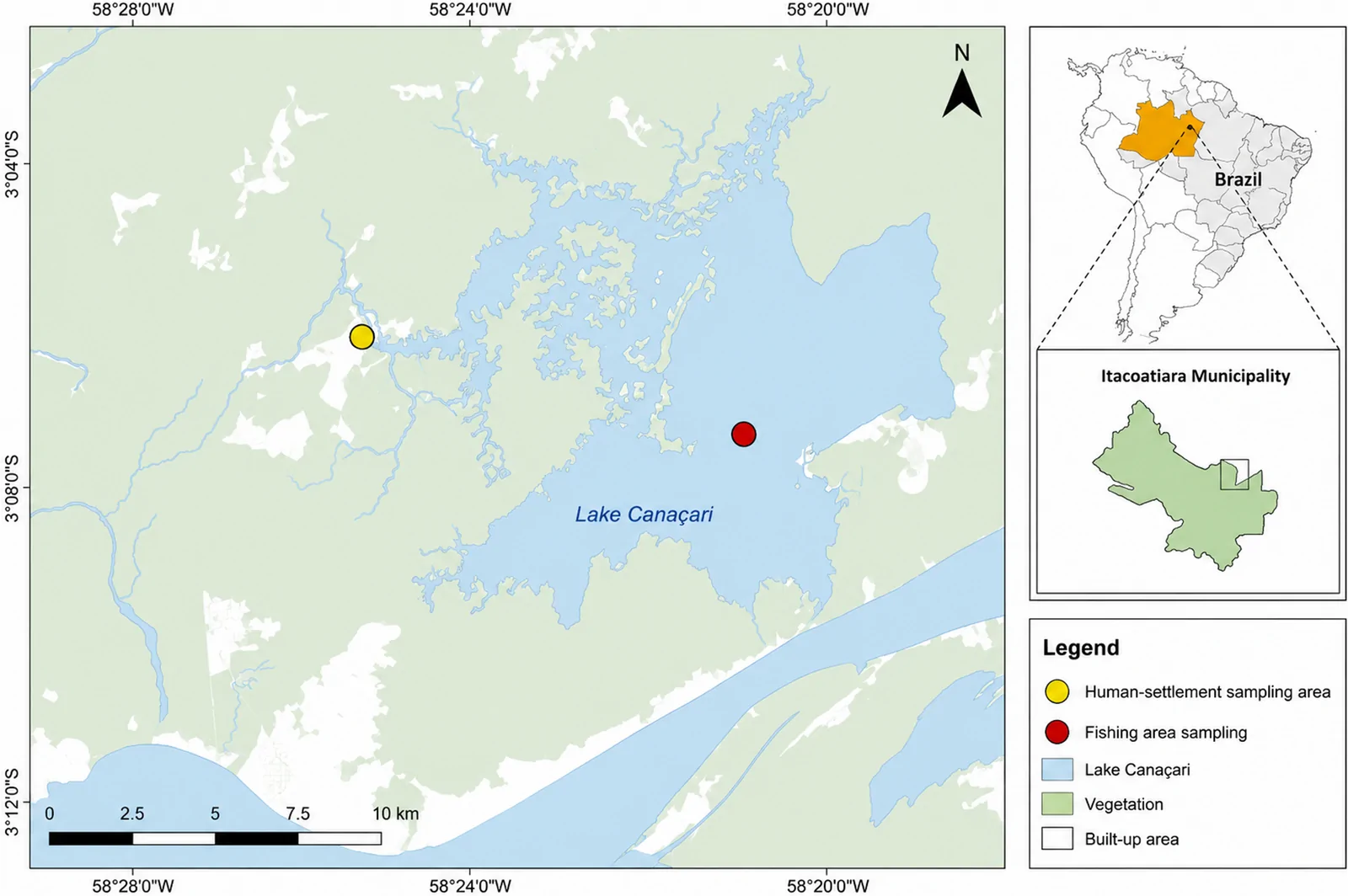
Study area in Lake Canaçari, Central Amazon, Brazil, showing the locations of the two sampling areas: the human-settlement area and the fishing area. The inset maps indicate the location of the study area within Amazonas State and Brazil

Samples were collected in October 2022 during the Amazonian dry season, corresponding to the low-water phase (Marengo & Espinoza, 2016). During this period, lower water levels generally reduce water exchange between floodplain lakes, adjacent channels, and the main river, resulting in reduced hydrological connectivity (Fassoni-Andrade et al., 2021). To compare microplastic levels, two sampling areas representing different predominant land-use characteristics were selected based on qualitative field observations: a fishing area and an area with human settlements. The fishing area (03°03′50.2″ S, 058°20′40.1″ W) was characterized by recurrent artisanal fishing activity, including the presence of boats and fishing gear such as nets and lines, as well as regular use by local fishers. The area with human settlements (03°02′39.8″ S, 058°25′35.5″ W) was characterized by the presence of riverside dwellings, visible solid-waste disposal, and direct domestic use of the water body (Fig. 1). These classifications were based on qualitative field observations, as quantitative indicators of anthropogenic pressure, such as distance to dwellings, number of boats, or waste density, were not systematically recorded during sampling. Seven water and sediment samples were collected in each area. Sampling points were distributed along the margins, with 10 m spacing between points.

Surface water collection was performed based on a methodology adapted from He et al. (2021). At each sampling point, 6 L of water were collected, consisting of three 2 L subsamples obtained from the near-surface water layer (0–30 cm). This depth range was selected to characterize microplastic contamination in surface waters, consistent with surface-water sampling approaches previously applied in freshwater lakes (Jiang et al., 2018). Samples were vacuum-filtered using Whatman paper filters (5.0 μm pore size). After filtration, the filters were individually placed in Petri dishes and kept at room temperature for 24 h for drying prior to subsequent analyses.

At each sampling point, 150 g of surface sediment were collected from the upper 5 cm using a stainless-steel spatula. Samples were dried in an oven at 70 °C for 72 h. For sediment samples only, 60 g of dry sediment were subsequently transferred to beakers and subjected to density separation with 200 mL of saturated NaCl solution (density 1.2 g·cm⁻^3^). The suspension was manually stirred for 1 min and allowed to settle for 5 min for sedimentation. The supernatant was carefully recovered, and the procedure was repeated three times for each sample to maximize extraction of any microplastic particles. The recovered material was subjected to organic matter digestion with 5 mL of 30% H₂O₂ (v/v) for 24 h at room temperature (Zhao et al., 2018; Oliveira et al., 2023). Subsequently, the solution was vacuum-filtered using Whatman paper filters (5.0 μm). The filters were placed in Petri dishes and dried for 24 h at room temperature prior to analysis.

During sampling, the team wore cotton clothing and blank controls were conducted in all sampled areas (Prata et al., 2021). Atmospheric contamination by microplastics was monitored through the exposure of Petri dishes containing filter papers, which were kept open during field procedures and sample processing. Samples were stored in glass containers previously rinsed with filtered water, avoiding the use of plastic materials. In the laboratory, cotton lab coats and glass, aluminum, or paper materials were used. Benches and equipment were cleaned with cotton cloths moistened with filtered water, and air circulation was minimized during sample handling. NaCl and H₂O₂ solutions were previously filtered. A total of 16 blank controls were analyzed for contamination monitoring and quality control, including 14 field blanks and 2 laboratory blanks. One blue fiber was detected across the field blanks, whereas two transparent fibers were detected across the laboratory blanks. Overall, the three particles detected in the blank controls corresponded to approximately 1.5% of the number of particles detected in the environmental samples, indicating that background contamination was low relative to the particle abundance observed in the environmental samples.

### Microplastic identification

Surface water and sediment samples were analyzed using a stereomicroscope (Leica, EZ4) with magnifications ranging from 13 × to 56 ×. Images of the particles were captured using a digital camera (Moticam 2300, 3.0 megapixels). Each particle was measured for maximum length (μm). Although 5 μm filters were used during sample processing, the minimum particle size considered for reliable visual identification and measurement by stereomicroscopy was 40 μm. Microplastic size was categorized as follows: 40–200 μm, 200–500 μm, 500–1,000 μm, 1,000–3,000 μm, and 3,000–5,000 μm. Within this analytical range, the smallest particle observed was 47 μm in sediment samples and 115 μm in surface-water samples. Particle shapes were classified as fibers or fragments, and colors were categorized as blue, green, or red. Each microplastic particle was counted and photographed. The physical characterization of the particles followed the protocol established by Lusher et al. (2020), minimizing the counting of false positives, i.e., the misclassification of natural particles as microplastic particles.

Microplastics obtained during the extraction steps were analyzed using Fourier-transform infrared spectroscopy (FTIR) and Raman spectroscopy. Approximately 8% of the visually identified particles were selected for FTIR and/or Raman spectroscopy, prioritizing samples with higher particle abundance. Therefore, the spectroscopic characterization represents the analyzed subset rather than the entire assemblage of visually identified particles. FTIR spectra were acquired using a spectrometer (Bruker, Alpha) equipped with an ATR module (Platinum) and diamond crystal, within the range of 400–4000 cm⁻^1^, with a KBr beamsplitter and a high-sensitivity DLATGS detector. Measurements were performed with 64 scans for the sample and 64 scans for the background, using a spectral resolution of 4 cm⁻^1^. Raman spectra were obtained using two instruments: (i) a Micro-Raman spectrometer (Renishaw inVia) (λ = 532, 633, and 785 nm), using a 50 × objective lens and optical imaging with 5 × or 50 × objective lenses, depending on the material; and (ii) a spectrometer (Witec 600-VIS) coupled to a confocal Raman microscope (Alpha300-R), with 633 nm excitation and a 50 × objective lens. The choice of excitation radiation was based on the response of pigments present in the plastics to the incident radiation.

### Statistical analyses

Mean microplastic concentrations in surface water and sediments between sampling areas were evaluated using the t-test. To compare microplastic size in surface water and sediments between sampling areas, the Mann–Whitney U test was applied. All statistical analyses were performed in the R environment (R Core Team, 2024), adopting a significance level of *p* < 0.05.

## Results and discussion

### Microplastics in surface water

Microplastics were detected in surface-water samples from both fishing and human-settlement areas of Lake Canaçari. Concentrations ranged from 0 to 1,000 microplastics·m⁻^3^, with a mean of 405 ± 298 microplastics·m⁻^3^. No microplastic particles were detected in one individual sample. The reason for this result cannot be determined from the present dataset and may reflect local spatial variability in particle distribution. The concentrations observed in Lake Canaçari were within the range reported for other aquatic systems (Table 1). However, comparisons among lakes should be interpreted cautiously because differences in sampling volume, sampling devices, mesh or filter pore size, and minimum particle-size thresholds can substantially influence reported microplastic concentrations (Cowger et al., 2020). Studies using finer meshes or lower analytical size thresholds are more likely to retain and identify smaller particles, potentially resulting in higher reported concentrations than studies using coarser sampling methods. Therefore, part of the variability among lakes may reflect methodological differences rather than environmental contamination alone.

**Table 1:** Worldwide distribution of microplastics in lake surface waters

Lake Name	Location	Sampling Method	Sample Volume	Minimum size threshold/mesh-filter size (μm)	Concentration (microplastics·m⁻^3^)	Study
Phewa Lake	Nepal	Steel bucket; filtration after pre-sieving	5 L	75 μm	1,510–2,960	Malla-Pradhan et al. (2022)
Gehu Lake	China	Submersible pump; filtration	–	Not reported	1,510–22,220	Xu et al. (2021)
Kolavai Lake	India	Steel bucket; mesh filtration	30 L	37 μm	6,100 ± 2,500	Rajeswari et al. (2023)
Nainital Lake	India	Steel bucket; sieving	–	20 μm	8,600–56,000	Jain et al. (2024)
Wabu Lake	China	Steel sampler	20 L	Not reported	13,700 ± 6,560	Chen et al. (2024)
Urban Lakes	Vietnam	Steel bucket; plankton-net filtration	100 L	300 μm (80 μm mesh)	643.8 ± 87.5–2,145.2 ± 322.6	Tran-Nguyen et al. (2024)
Lake Victoria	Africa	Trawl/manta net	–	300 μm	0.73	Egessa et al. (2020)
Lake Mainit	Philippines	Glass bottle	15 L	Not clearly reported	313.33 ± 252.11	Arcadio et al. (2025)
Poyang Lake	China	Steel sampler; filtration	20 L	50 μm	5,000–34,000	Yuan et al. (2019)
Luruaco Lake	Colombia	Surface-water collection; net filtration	20 L	23 μm	730–1,470	Rojas-Luna et al. (2023)
Guaíba Lake	Brazil	Trawl/manta net	35 m^3^	60 μm	11.9 ± 0.6–61.2 ± 6.1	Bertoldi et al. (2021)
Lake Canaçari	Brazil	Surface-water sampler; vacuum filtration	6 L	40 μm	405 ± 298	Present study

**Fig. 2:**
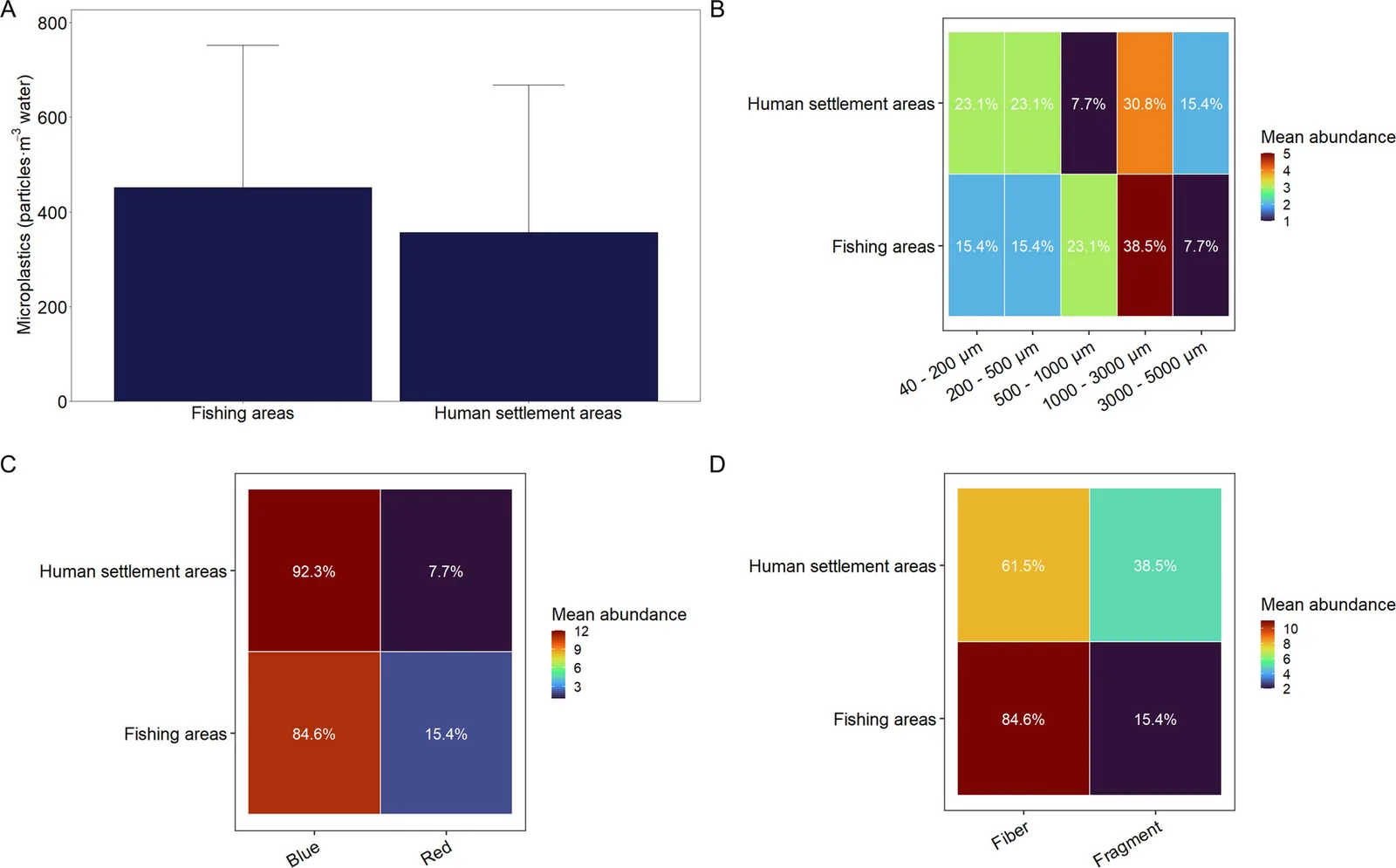
Microplastic distribution in surface waters of Lake Canaçari. (**A**) Mean concentration by sampling area; error bars indicate ± standard deviation. (**B**) Size distribution of microplastic particles. (**C**) Color distribution of microplastic particles. (**D**) Shape distribution of microplastic particles. No significant differences were detected between sampling areas in microplastic concentration (*p* = 0.5704) or particle size (*p* = 0.801)

No significant difference was observed in microplastic concentrations in surface water between the human-settlement and fishing areas (t = −0.5835; df = 11.985; *p* = 0.5704) (Fig. 2A). However, given the relatively small sample size (n = 7 per area), the absence of a significant difference should be interpreted cautiously, as the study may have limited ability to detect small or moderate spatial differences. Therefore, the present results indicate no detectable difference in microplastic concentrations between the sampled areas under the conditions evaluated, rather than demonstrating spatial homogeneity. Beyond the comparison between areas, microplastic distribution in aquatic systems may be influenced by particle properties such as density, shape, and polymer composition (Akdogan & Güven, 2025), as well as by biofilm formation, association with particulate organic matter (Zettler et al., 2013), retention by aquatic macrophytes (Guimarães et al., 2024), and local hydrodynamic conditions such as depth, substrate type, and flow velocity (Chen et al., 2025).

In low-energy lacustrine environments, transport tends to be slower, favoring the deposition and localized accumulation of particles. In contrast, regimes with higher discharge and turbulence promote greater dispersion and reduce sedimentation (Chen et al., 2025). As an Amazonian floodplain system, Lake Canaçari is subject to seasonal changes in hydrological conditions that may influence microplastic transport and redistribution. However, because sampling was restricted to the dry season, these seasonal dynamics could not be directly evaluated in the present study. The likely sources of the particles are mainly associated with local anthropogenic activities. In fishing areas, the main contributors include the use of fishing gear, disposal of plastic packaging, and degradation of paints and boat materials (Azaaouaj et al., 2025). In areas with human settlements, the predominant sources include inadequate solid waste disposal, rural activities, and the discharge of untreated effluents (Dong et al., 2025). This scenario is aggravated by the lack of sanitation infrastructure in the Amazon region, especially regarding wastewater collection and treatment and solid waste management (Morais et al., 2024), which maintains the continuous input of these particles into water bodies.

The size of the microplastic particles ranged from 115 to 3,615 μm, with a mean of 1,241 ± 1,124 μm. No difference in particle size was observed in surface water between fishing and human-settlement areas (W = 79; *p* = 0.801) (Fig. 2B), indicating a broad overlap in size classes within the system. The presence of a wide size range suggests the action of secondary fragmentation processes influenced by ultraviolet radiation, mechanical abrasion, and hydrodynamics (Hebner & Maurer-Jones, 2020). Smaller particles tend to exhibit greater mobility and longer residence time in the water column (Warrier et al., 2022), especially during periods of lower hydrodynamic energy, such as the dry season, when their retention may be favored (Russell et al., 2023). This behavior increases the likelihood of interaction and ingestion by aquatic organisms in Lake Canaçari, with potential ecological effects (Azevedo et al., 2024).

Microplastic particles were predominantly blue and red in both sampled areas (Fig. 2C). Regarding morphology, fibers and fragments were identified, with fibers predominating across all areas (Fig. 2D). The higher abundance of fibers in the fishing area suggests a strong influence of fishing activities using synthetic polymers, frequently associated with blue fibers (Azevedo et al., 2024), which are used in gillnets, reels, cast nets, and other fishing gear (Guimarães et al., 2026). The predominance of fibers, especially blue and red ones, is also ecologically relevant, as such particles may be mistaken for prey by aquatic organisms, increasing the risk of accidental ingestion and its associated impacts (Guimarães et al., 2023).

### Microplastics in sediments

**Fig. 3:**
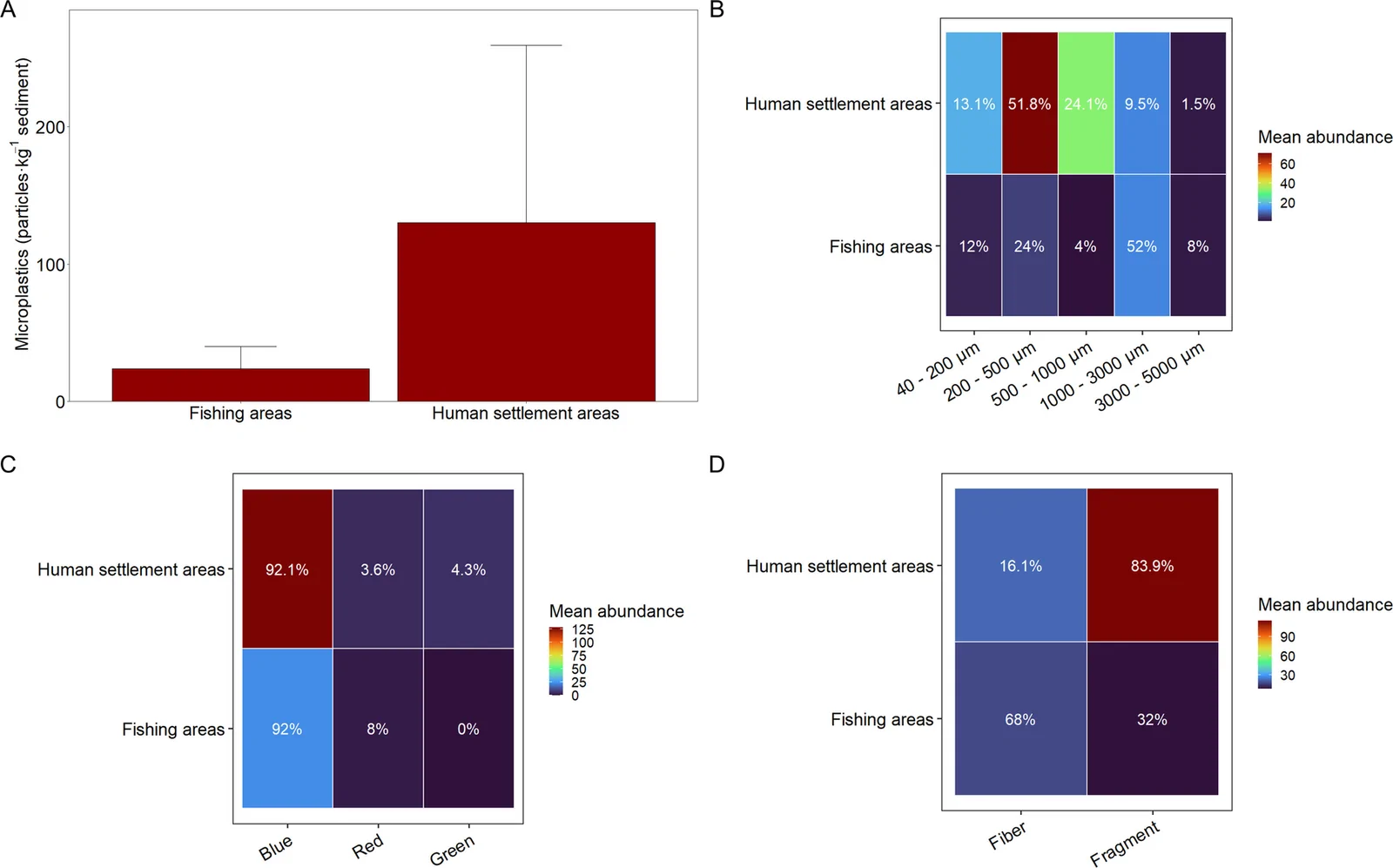
Microplastic distribution in the sediments of Lake Canaçari. (**A**) Mean concentration of microplastics by sampling area; error bars represent ± standard deviation. (**B**) Size distribution of microplastic particles. (**C**) Color distribution of microplastic particles. (**D**) Shape distribution of microplastic particles. No significant difference was detected in microplastic concentration between areas (*p* = 0.0712), whereas particle size differed significantly between areas (*p* = 0.0029)

Microplastics were detected in the sediments of Lake Canaçari, with concentrations ranging from 7 to 307 particles·kg⁻^1^ of dry sediment and a mean of 77 ± 104 particles·kg⁻^1^. No significant difference was observed between sampling areas (Fig. 3A) (t = 2.175; df = 6.192; *p* = 0.0712). The concentrations recorded were generally lower than those reported for several other lacustrine systems (Table 2). However, comparisons among studies should be interpreted cautiously because differences in sediment sampling depth, sample processing, density-separation methods, filtration procedures, and minimum particle size can substantially affect microplastic recovery and reported concentrations (Cowger et al., 2020). Therefore, the lower concentrations observed in Lake Canaçari may reflect both environmental characteristics and methodological differences among studies.

**Table 2:** Worldwide distribution of microplastics in lake sediments

Lake Name	Location	Sampling/processing method	Minimum size (μm)	Concentration (microplastics·kg⁻^1^)	Study
Bolsena Lake	Italy	Shore sediment; NaCl separation	Not reported	112 ± 32	Fischer et al. (2016)
Chiusi Lake	Italy	Shore sediment; NaCl separation	Not reported	234 ± 85	Fischer et al. (2016)
Taihu Lake	China	Benthic sediment; NaCl separation	Not reported	893.48 ± 245.74	Zhang et al. (2021)
Landscape Lake	China	Sediment core (0–5 cm); ZnCl₂ separation	20	8,200	Yan et al. (2022)
Aha Lake	China	Sediment cores; 5-cm intervals	Not reported	538 ± 429	Gao et al. (2023)
Caohai Lake	China	Lake sediment sampling	Not reported	4,410 ± 1,635–4,783 ± 1,892	Wu et al. (2023)
Qinghai Lake	China	Surface sediment (0–5 cm); ZnCl₂ separation; filtration	20	393 ± 457	Jiang et al. (2022)
Phewa Lake	Nepal	Lakeshore sediment; density separation; filtration	Not reported	100.5 ± 58.6	Malla-Pradhan et al. (2023)
Ashtamudi Lake	India	Sediment sampling; H₂O₂ digestion; NaCl separation	Not reported	3 ± 1.69	Devi et al. (2024)
Lake Canaçari	Brazil	Surface sediment (0–5 cm); NaCl separation; H₂O₂ digestion; vacuum filtration	40	77 ± 104	Present study

Microplastic distribution in the sediments of Lake Canaçari may also be influenced by hydrodynamic and depositional processes. Precipitation and resuspension events regulate the transport and accumulation of particles in the benthic zone (Jiang et al., 2022). Under these conditions, sediment can act as a retention compartment, particularly under low-energy conditions. In addition, interactions with fine particles such as clays and organic matter can favor aggregate formation, increasing the apparent density of microplastics and promoting their deposition (Chubarenko et al., 2020).

The size of microplastic particles ranged from 47 to 4,720 μm (678 ± 751 μm), with a significant difference between areas (W = 1068.5; *p* = 0.0029). Smaller to intermediate particles predominated in the human-settlement area, whereas intermediate to larger particles were more frequent in the fishing area (Fig. 3B). This difference may reflect a combination of source characteristics and size-dependent transport and deposition processes. Smaller particles, because of their higher surface-to-volume ratio, may interact more readily with fine sediments and organic matter and become incorporated into organo-mineral aggregates, favoring their retention in sediments (Kinigopoulou et al., 2022; Muñoz et al., 2021). In contrast, the greater occurrence of intermediate to larger particles in the fishing area may also reflect local inputs from the degradation and fragmentation of fishing-related plastic materials (Devi et al., 2024; Ramos et al., 2025).

A comparable difference in particle size was not detected in surface water. In this compartment, greater particle mobility and hydrodynamic redistribution may reduce the spatial differentiation associated with local sources or size-dependent deposition (Chen et al., 2025). Thus, sediment may preserve spatial differences in particle size more effectively than surface water. However, because particle settling velocities and local hydrodynamic conditions were not directly measured, these mechanisms should be considered plausible interpretations rather than confirmed processes.

Particles were predominantly blue, followed by red and green (Fig. 3C). The preservation of coloration in sediments suggests pigment stability and potential use as an indirect indicator of aging (Zhao et al., 2022). Regarding morphology, fibers and fragments were identified, with a distinct spatial pattern (Fig. 3D). Fragments predominated in the human occupation area, while fibers were more abundant in the fishing area, reflecting differences in their sources. In fishing areas, fibers are associated with the wear of synthetic nets, lines, and ropes (Devi et al., 2024), whereas in the human occupation area, fragments indicate the fragmentation of domestic waste (Kabir et al., 2022). In comparison with other tropical systems (Malla-Pradhan et al., 2023), the predominance of fibers and fragments is consistent. These morphological differences may reflect contributions from different local activities; however, because source-specific differences in particle morphology were not statistically tested, this interpretation should be considered tentative.

**Fig. 4:**
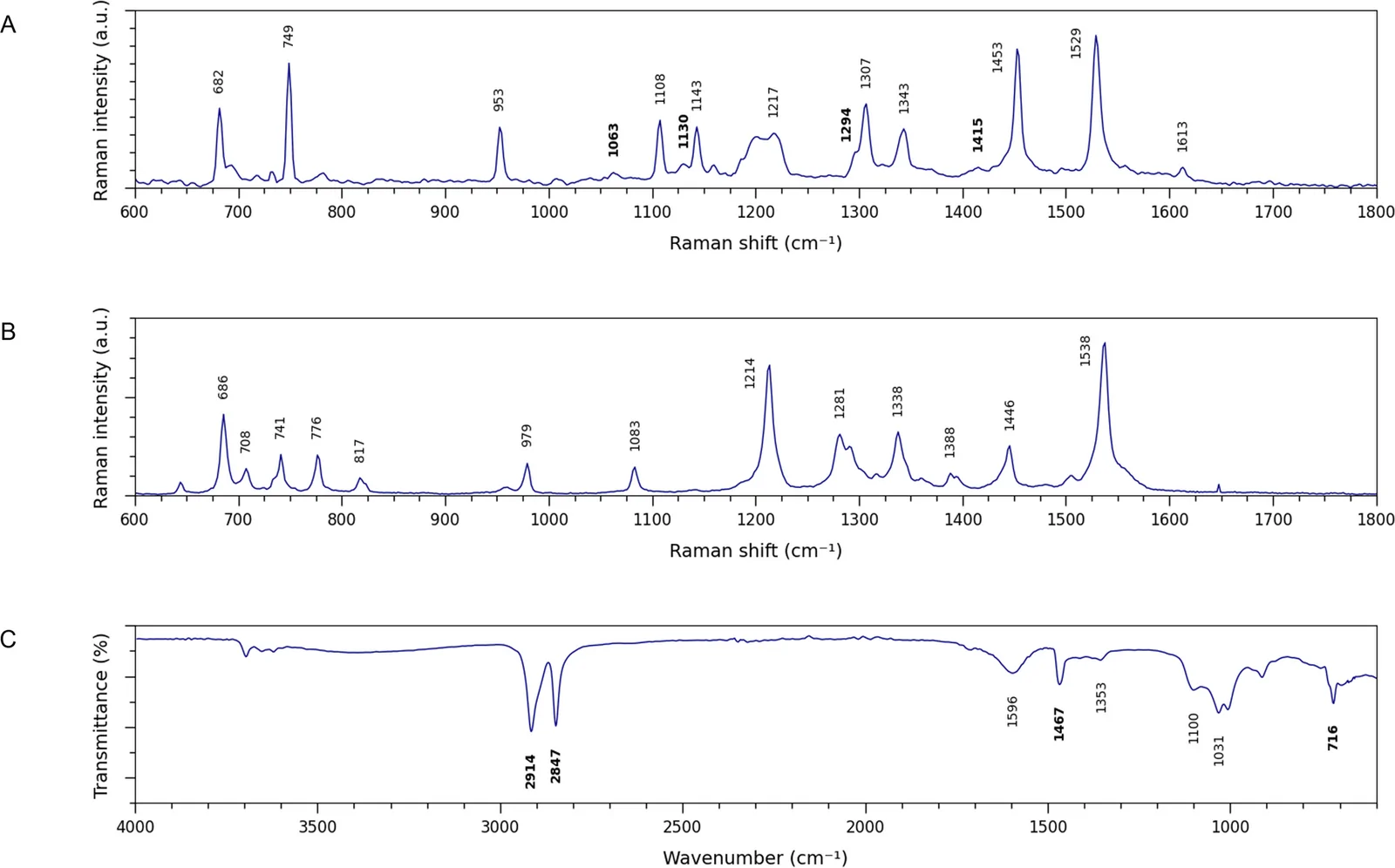
Polymeric characterization of microplastics in surface water and sediment from Lake Canaçari. (**A**) Raman spectrum of polyethylene with Pigment Blue 15 dye. (**B**) Raman spectrum of a fragment containing Pigment Green 7 dye. (**C**) FTIR spectra of polyethylene mixed with cellulose. Bold numbers in the spectra correspond to polyethylene bands

Spectroscopic analysis using FTIR and Raman indicated a dominance of polyethylene-based microplastics, identified by both techniques. The observed sample loss was associated with the presence of adhered dyes and the advanced degradation state of the particles, factors that compromised spectral quality and limited the identification of other polymers. One Raman spectrum showed characteristic bands of polyethylene associated with the dye Pigment Blue 15 (Scherrer et al., 2009) (Fig. 4A), with signals at 1063 cm⁻^1^ (C–C asymmetric stretching), 1130 cm⁻^1^ (C–C symmetric stretching), 1294 cm⁻^1^ (CH₂ twisting), and 1415 cm⁻^1^ (CH₂ scissoring) (Nava et al., 2021; Umurhan et al., 2025). Additionally, using the same technique, the dye Pigment Green 7 was identified in one of the analyzed fragments (Fig. 4B) (Chaplin & Clark, 2016; Barba Castagnaro et al., 2025); however, the high intensity of its signal prevented the identification of the underlying polymer matrix. The FTIR spectrum of another analyzed fragment showed characteristic bands of polyethylene associated with cellulose (Fig. 4C), with bands at 2914 cm⁻^1^ (asymmetric stretching of CH₂ groups), 2847 cm⁻^1^ (symmetric stretching of CH₂ groups), 1467 cm⁻^1^ (CH₂ scissoring), and 716 cm⁻^1^ (CH₂ rocking) for the synthetic polymer (Fan et al., 2021; Jung et al., 2018).

The predominance of polyethylene-based fragments suggests contributions from sources associated with daily use, such as packaging, bags, household items, and agricultural materials (Akdogan et al., 2023). Although this polymer can also occur as primary microplastics, the overall results indicate a predominance of secondary origin in Lake Canaçari, resulting from the environmental degradation and fragmentation of larger plastic debris through processes such as UV exposure, weathering, and mechanical abrasion (An et al., 2020). This process may be particularly relevant in human-settlement areas, where improper waste disposal can favor the accumulation and subsequent degradation of plastic materials (Drabinski et al., 2023). The presence of Pigment Blue 15, a widely used organic pigment, may enhance the photodegradation of plastic under UV radiation, promoting fragmentation and the release of potentially toxic compounds (Lopes et al., 2024; Saron et al., 2006). Additionally, the detection of Pigment Green 7, previously reported in microplastic samples (Schymanski et al., 2018) and widely used in paint and coating formulations (Scherrer et al., 2009), indicates a possible anthropogenic source within the Lake Canaçari region.

## Study limitations and future perspectives

This study provides a baseline assessment of microplastic contamination in Lake Canaçari; however, some limitations should be considered. Sampling was conducted during a single campaign in the dry season, with restricted spatial coverage and land-use categories defined mainly from qualitative field observations. Surface-water sampling was limited to the upper 30 cm, whereas sediment sampling was restricted to the upper 5 cm, preventing assessment of potential vertical gradients in surface waters and historical accumulation in deeper sediments. In addition, site-specific hydrodynamic and environmental variables were not measured. Future studies should therefore expand the spatial coverage, incorporate quantitative indicators of anthropogenic pressure, include contrasting hydrological periods and multiple sampling depths, use deeper sediment sampling or sediment cores, and measure relevant environmental and hydrodynamic variables.

Methodological limitations should also be considered. For sediment samples, density separation using saturated NaCl solution (1.2 g·cm⁻^3^), despite being a widely used technique for separating microplastics, may have reduced the recovery of higher-density polymers, potentially underestimating their abundance and polymer diversity in this compartment. This limitation does not apply to surface-water samples, which were directly filtered without density separation. In addition, approximately 8% of the visually identified particles were subjected to FTIR and/or Raman spectroscopy. Therefore, the polymer composition reported here represents the spectroscopically analyzed subset rather than the entire particle assemblage. Particle degradation, adhered pigments, spectral interference, and sample loss also limited polymer identification in some cases. Future studies should increase the proportion of particles subjected to spectroscopic confirmation and consider higher-density separation solutions, such as Zinc chloride (ZnCl₂) or Sodium iodide (NaI) for sediments to improve the recovery of a broader range of polymers. Together, these improvements will provide a more comprehensive understanding of the spatial and temporal distribution, sources, transport, and accumulation of microplastics in Amazonian floodplain systems.

## Conclusion

This study demonstrates the occurrence of microplastics in Lake Canaçari in both surface water and sediments, confirming that this Amazonian floodplain system is affected by microplastic contamination. No significant difference in microplastic concentrations was detected between the fishing and human-settlement areas during the dry season; however, given the relatively small sample size, this result should not be interpreted as evidence of spatial homogeneity. Blue fibers predominated in surface water in both areas, whereas fragments were more frequent in sediments from the human-settlement area and fibers predominated in the fishing area. These compositional patterns may reflect contributions from different local activities, although source attribution should be considered tentative. The identification of polyethylene and copper phthalocyanine pigments further indicates anthropogenic inputs to the system. Overall, the results suggest that Lake Canaçari may function as a transport pathway, redistribution zone, and retention compartment for microplastics. However, because sampling was restricted to the dry season, seasonal studies are needed to determine how these processes vary throughout the hydrological cycle. These findings highlight the occurrence and potential retention of microplastics in Amazonian floodplain systems and reinforce the need for monitoring strategies that incorporate seasonal hydrological variability.

## Data Availability

Data will be made available upon reasonable request.
